# Redox modulation contributes to the antidepressant-like and neuroprotective effects of 7-chloro-4-(phenylselanyl)quinoline in an Alzheimer’s disease model

**DOI:** 10.1080/13510002.2026.2626641

**Published:** 2026-02-16

**Authors:** Renata L. De Oliveira, Mikaela P. Pinz, Guilherme T. Voss, Karline Da C. Rodrigues, Ane G. Vogt, Victor Dos S. Barboza, Rodrigo De A. Vaucher, Janice L. Giongo, Ariana Silveira Lima, Diego Alves, Caroline B. Quines, Eduarda M. Fidelis, Simone Pinton, Lucielli Savegnago, Cristiane Luchese

**Affiliations:** a Graduate Program in Biochemistry and Bioprospecting, Cognitive and Behavioral Neuroscience Research Laboratory (LaNeC), Center for Chemical, Pharmaceutical and Food Sciences (CCQFA), Federal University of Pelotas, Pelotas, Brazil; b Graduate Program in Biotechnology, Neurobiotechnology Research Group (GPN), Center for Technological Development (CDTec), Federal University of Pelotas, Pelotas, Brazil; c Department of Biochemistry, Institute of Chemistry, University of São Paulo, São Paulo, Brazil; d Graduate Program in Biochemistry and Bioprospecting, Research Laboratory in Biochemistry and Molecular Biology of Microorganisms (LaPeBBiOM), Center for Chemical, Pharmaceutical and Food Sciences (CCQFA), Federal University of Pelotas, Pelotas, Brazil; e Graduate Program in Chemistry, Laboratory of Clean Organic Synthesis (LASOL), Center for Chemical, Pharmaceutical and Food Sciences (CCQFA), Federal University of Pelotas, Pelotas, Brazil; f Graduate Program in Biochemistry, Research Group in Biochemistry and Toxicology in Eukaryotes, Federal University of Pampa, Uruguaiana, Brazil

**Keywords:** Comorbidity, depression, oxidative stress, neuroinflammation, redox homeostasis, selenium, neuroprotection, antioxidant

## Abstract

**Objectives:**

Alzheimer’s disease (AD) is characterized by cognitive impairment and neuropsychiatric disturbances, including depression, both tightly linked to redox imbalance and neuroinflammatory activation. This study investigated whether the selenium-containing compound 7-chloro-4-(phenylselanyl)quinoline (4-PSQ) mitigates behavioral and biochemical alterations in a β-amyloid (Aβ)-induced mouse model of AD through modulation of redox-regulated pathways.

**Methods:**

Male Swiss mice received intracerebroventricular Aβ (25–35) or saline (3 µL/site) and were treated orally for seven days with 4-PSQ (1 mg/kg), paroxetine (1 mg/kg), or donepezil (1 mg/kg). Depressive-like behavior and memory performance were assessed, followed by determination of plasma corticosterone, reactive species levels, lipid peroxidation, antioxidant enzyme activities, neuroinflammatory mediators, and acetylcholinesterase (AChE) activity in the hippocampus and prefrontal cortex of mice.

**Results:**

4-PSQ significantly reversed Aβ-induced depressive behavior and memory impairment. The compound normalized plasma corticosterone levels, reduced reactive species and lipid peroxidation, and restored antioxidant enzyme activity. It also decreased the expression of inflammatory markers while regulating AChE activity, indicating concomitant modulation of redox, neuroimmune, and cholinergic pathways.

**Conclusion:**

By restoring redox homeostasis and attenuating neuroinflammatory responses, 4-PSQ effectively counteracted behavioral and biochemical disruptions associated with Aβ toxicity. These findings support 4-PSQ as a promising selenium-based therapeutic candidate targeting redox-driven features of AD, including comorbid depression and cognitive decline.

## 1. Introduction

Alzheimer’s disease (AD) is a progressive neurodegenerative disorder marked by cognitive decline and neuronal loss in brain regions involved in memory and emotional regulation [1]. Its main pathological hallmarks are β-amyloid (Aβ) plaques and intracellular neurofibrillary tangles of hyperphosphorylated tau [2]. Depression is the most frequent neuropsychiatric comorbidity of AD, affecting up to 50% of patients and worsening disease progression [3].

The molecular mechanisms linking depression and AD include hypothalamic–pituitary–adrenal (HPA) axis hyperactivation, oxidative stress, neuroinflammation, and cholinergic dysfunction [3,4]. Excessive production of reactive species (RS) and impaired antioxidant defenses promote Aβ-induced neurotoxicity and neuronal vulnerability [5]. Restoring redox homeostasis can mitigate cognitive and emotional disturbances associated with AD [6,7].

Current pharmacological strategies offer limited efficacy in treating depression comorbid with AD because they do not target oxidative and inflammatory mechanisms [4,8]. Thus, molecules capable of modulating redox signaling and neuroinflammatory responses represent promising candidates.

Selenium, an essential trace element incorporated into glutathione peroxidase (GPx) and thioredoxin reductases, plays a pivotal role in neuronal redox balance [9,10]. Inspired by these selenoproteins, organoselenium compounds have emerged as redox-active molecules with antioxidant and neuroprotective properties [5,9]. Among them, 7-chloro-4-(phenylselanyl)quinoline (4-PSQ) shows neuroprotective, anticholinesterase, and antidepressant-like effects in different models [11–17]. Its selenium moiety imparts redox activity, enabling RS scavenging and modulation of oxidative, inflammatory, and cholinergic pathways central to AD pathophysiology.

Given the shared oxidative and inflammatory mechanisms between depression and AD, this study aimed to evaluate the neuroprotective and antidepressant effects of 4-PSQ in an Aβ (25–35)-induced murine model of AD, emphasizing redox modulation as a key mechanism.

## 2. Materials and methods

Detailed experimental procedures of this study are available in the *Supplementary Material*.

### 2.1. Animals and ethical approval

Male Swiss mice (25–35 g, 60 days old) from the local breeding colony of the Federal University of Pelotas (UFPel) were used. All procedures were approved by the Ethics Committee on Animal Use of the Federal University of Pelotas (CEUA/UFPel, protocol number 1974–2016). All behavioral assessments were conducted during the light phase, and every effort was made to minimize animal discomfort and reduce the number of animals used. At the end of the experimental protocol, mice were deeply anesthetized with isoflurane until loss of reflexes, followed by euthanasia through cervical dislocation, in accordance with the AVMA Guidelines for the Euthanasia of Animals and institutional ethical approval.

### 2.2. Chemicals

**Figure 1.**
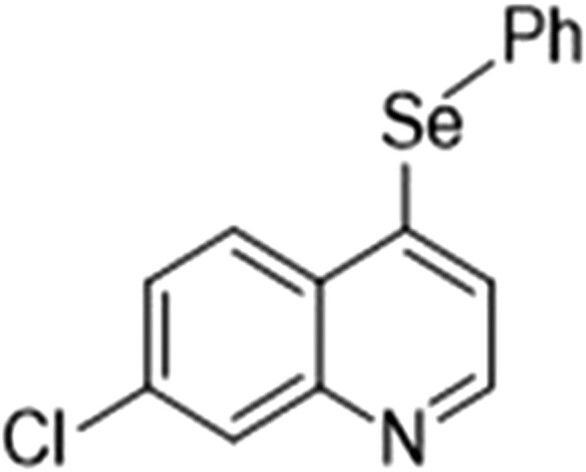
Chemical structure of 7-chloro-4-(phenylselanyl) quinoline (4-PSQ).

The 4-PSQ (Fig. 1) was prepared according to the literature Duarte et al [18], through nucleophilic substitution between 7-chloroquinoline and phenylselenolate generated in situ. The chemical purity of the compound (99.9%) was determined by gas chromatography – mass spectrometry (GC/MS). Chemicals and reagents were obtained from Sigma-Aldrich (St. Louis, MO, USA) with analytical-grade purity. β-Amyloid peptide fragment 25–35 (Aβ 25–35, cat. A4559, Sigma-Aldrich) was dissolved in sterile filtered water and incubated at 37°C for 4 days to allow aggregation prior to intracerebroventricular (i.c.v.) administration. 4-PSQ, paroxetine, and donepezil were dissolved in canola oil and administered intragastrically (i.g.) at 1 mg/kg (10 mL/kg). The 4-PSQ dose was based on previous studies demonstrating its efficacy and safety under similar conditions.

### 2.3. Experimental design

Mice were randomly assigned to six groups (7 animals/group). On day 1, animals received oral treatments: canola oil (10 mL/kg) for the Sham and Aβ groups; 4-PSQ (1 mg/kg, i.g.) for the 4-PSQ and Aβ + 4-PSQ groups; and paroxetine (1 mg/kg, i.g.) or donepezil (1 mg/kg, i.g.) for the Aβ + paroxetine and Aβ + donepezil groups, respectively. Thirty minutes later, animals in the Aβ, Aβ + 4-PSQ, Aβ + paroxetine, and Aβ + donepezil groups received intracerebroventricular administration of aggregated Aβ (25–35) (3 nmol/3 µL per site), whereas the Sham and 4-PSQ groups received saline (3 µL per site). Injections were performed under isoflurane anesthesia using a 28-gauge needle following stereotaxic coordinates described by Haley and McCormick [19].

**Figure 2.**
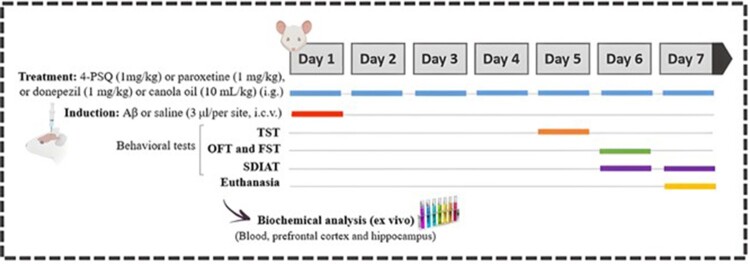
Scheme of experimental protocol. Thirty minutes before starting intragastric (i.g.) treatments, mice received amyloid β-peptide (Aβ, fragment 25–35) in aggregated form or vehicle (saline), both intracerebroventricularly (i.c.v.). Treatments were performed every day, until the end of the experimental protocol. Between the fifth and seventh day of the protocol, the animals were submitted to behavioral tests: tail suspension test (TST), open field test (OFT), forced swimming test (FST) and step-down inhibitory avoidance tests (SDIAT). On the seventh- day, after the behavioral assessment, the animals were sacrificed for biochemical analyzes (ex vivo).

Treatments continued once daily throughout the 7-day protocol (Fig. 2). Behavioral assessments began on day 5. On day 7, after the final behavioral test, mice were anesthetized with isoflurane for blood collection by cardiac puncture, then euthanized for hippocampus and prefrontal cortex dissection for biochemical analyses.

It is worth noting that although six experimental groups were included in the behavioral analyses to validate the pharmacological model using established positive controls, biochemical and molecular evaluations were restricted to four groups (Sham, 4-PSQ, Aβ, and Aβ + 4-PSQ). This decision was based on the well-established mechanisms of paroxetine and donepezil in Alzheimer’s disease models. Therefore, additional ex vivo biochemical assessments in these groups were not considered necessary to address the main objective of this study, which was to investigate the redox-mediated neuroprotective mechanisms of 4-PSQ.

### 2.4. Behavioral tests

#### 2.4.1. Open-Field test (OFT)

General locomotor and exploratory activity were assessed to exclude motor impairment. Mice were placed in the center of the arena and evaluated for locomotion (crossings) and exploratory behavior (rearings) for 4 min [20].

#### 2.4.2. Tail suspension test (TST)

Depressive-like behavior was assessed according to Steru et al. [21]. Mice were suspended by the tail for 6 min, and immobility time was recorded. Reduced immobility reflects antidepressant-like behavior.

#### 2.4.3. Forced swimming test (FST)

Conducted according to Porsolt et al. [22]. Mice were placed in a cylinder filled with water (25 ± 1°C) for 6 min, and immobility time was quantified. Decreased immobility indicates antidepressant-like effects.

#### 2.4.4. Step-Down inhibitory avoidance test (SDIAT)

Long-term memory was evaluated following Sakaguchi et al. [23]. Mice received a footshock (0.5 mA, 2 s) upon stepping down from a platform during training. The test session occurred 24 h later without shock, and transfer latency was recorded (max 300 s). Longer latency indicates improved memory retention.

### 2.5. Biochemical analyses

#### 2.5.1. Sample preparation

On day seven of the experimental protocol, mice were anesthetized with isoflurane, and blood was collected from the heart ventricle using heparin as an anticoagulant. Plasma was obtained by centrifugation (900 × g, 15 min) and used for corticosterone quantification. The hippocampus and prefrontal cortex were immediately removed and homogenized in cold 50 mM Tris–HCl buffer (pH 7.4; 1:10 w/v). The homogenates were centrifuged (900 × g, 10 min, 4°C) to obtain the S1 fractions, which were used to evaluate oxidative stress parameters and antioxidant enzyme activity. For the evaluation of acetylcholinesterase (AChE), the tissues were homogenized separately in 0.25 M sucrose buffer (1:10 w/v) and centrifuged under the same conditions to obtain the supernatants for enzymatic activity analysis.

#### 2.5.2. Plasma corticosterone

Plasma corticosterone was quantified by the fluorometric method of Zenker & Bernstein [24], providing an index of HPA axis modulation.

#### 2.5.3. Oxidative stress parameters

RS generation was quantified using the dichloro-dihydro-fluorescein diacetate (DCFH-DA) fluorescence method as previously described by Loetchutinat et al. [25]. Lipid peroxidation was assessed through the thiobarbituric acid reactive substances (TBARS) assay following the protocol of Ohkawa et al. [26]. Results were expressed as fluorescence units (UF) for RS and as nmol malondialdehyde (MDA)/mg protein for TBARS.

#### 2.5.4. Antioxidant enzyme activity

Superoxide dismutase (SOD) activity was determined by its ability to inhibit epinephrine autoxidation [27]. GPx activity was measured by NADPH consumption in a coupled reaction system [28]. Values were normalized to protein content.

#### 2.5.5. Neuroinflammatory markers

mRNA expression of tumor necrosis factor-α (TNF-α) and interleukin-6 (IL-6) was quantified by real-time PCR using SYBR Green chemistry. Results were analyzed using the 2^−^ΔΔCT method and expressed relative to GAPDH. Additional methodological details are provided in the *Supplementary Material*.

#### 2.5.6. GFAP immunofluorescence

Immunofluorescence was performed using anti-GFAP primary antibody and appropriate fluorophore-conjugated secondary antibodies. Images were acquired with a fluorescence microscope, and quantification was conducted using ImageJ. Full technical details are described in the *Supplementary Material*.

#### 2.5.7. Acetylcholinesterase (AChE) activity

AChE activity was measured according to Ellman et al. [29] using acetylthiocholine as substrate, with absorbance recorded at 412 nm. Results were expressed as µmol substrate hydrolyzed/h/mg protein.

### 2.6. Statistical analysis

Data normality was assessed using the D’Agostino–Pearson test. All datasets showed normal distribution; therefore, parametric comparisons among groups were performed using one-way analysis of variance (ANOVA), followed by Tukey’s post hoc test. Results are presented as mean ± SEM, and statistical significance was set at *p* < 0.05. Analyses were conducted using GraphPad Prism (GraphPad Software, San Diego, CA, USA).

## 3. Results

### 3.1. 4-PSQ prevent against Aβ-induced memory impairment

**Figure 3.**
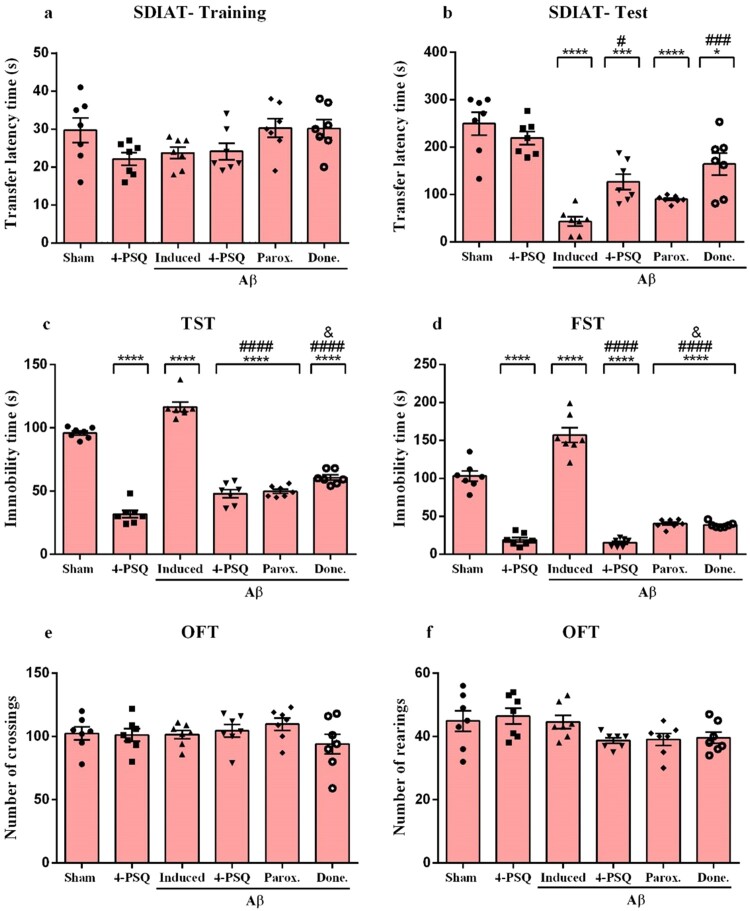
Effect of 7-chloro-4-(phenylselanyl)quinoline (4-PSQ) or paroxetine (Parox.) or donepezil (Done) in the behavioral changes induced by amyloid β (Aβ) peptide. (a) Training and (b) test at the Step-down inhibitory avoidance (SDIAT), (c) tail suspension test (TST), (d) forced swimming test (FST), (e) crossings and (f) rearings at the open field test (OFT). Values are expressed as mean ± standard error of the mean (S.E.M.) (*n* = 7). (*) denotes *p* < 0.05, (***) denotes *p* < 0.001 and (****) denotes *p* < 0.0001 when compared to the sham group. (#) denotes *p* < 0.05, (###) denotes *p* < 0.001 and (####) denotes *p* < 0.0001 when compared with the Aβ-induced group. (&) denotes denotes *p* < 0.05 when compared with the Aβ + 4-PSQ group (One-way ANOVA followed by the Tukey's test).

According to the results, in the training phase, there was no difference in the transfer latency time among groups (Fig. 3a). On the other hand, in the test phase, Aβ decreased (around 85%) the transfer latency time, when compared to the Sham group. 4-PSQ attenuated the reduction in transfer latency time similarly to donepezil, (Fig. 3b). On the other hand, the treatment with paroxetine did not change of transfer latency time in SDIAT. Treatment with 4-PSQ *per se* did not change of transfer latency time of mice. (ANOVA: *F*_(5,36)_ = 2.668, *p* < 0.0001 for training phase and ANOVA: *F*_(5,36)_ = 21.79, *p* < 0.0001 for test phase).

### 3.2. 4-PSQ prevent against Aβ-induced depressive-like behavior without altering locomotor and exploratory activity

The one-way ANOVA followed by Tukey's post-hoc test revealed that mice induced with Aβ showed an increase (around 18% in TST and 52% in FST) in duration of immobility time, when compared to the Sham group. The immobility time was attenuated by treatment with 4-PSQ, paroxetine and donepezil in the TST (Fig. 3c) and FST (Fig. 3d). 4-PSQ effect was superior to donepezil and paroxetine in attenuating the depressant-like behavior caused by Aβ. Treatment with 4-PSQ decreased *per se* the immobility time in TST and FST, when compared with the sham group. (ANOVA: *F*_(5,36)_ = 146.0, *p* < 0.0001 for TST and ANOVA: *F*_(5,36)_ = 115.6, *p* < 0.0001 for FST).

The data analysis of OFT showed no change in the number of crossings (ANOVA: *F*_(5,36)_ = 0.9207, *p* = 0.46798) and rearings (ANOVA: *F*_(5,36)_ = 2.466, *p* = 0.0508) after the treatments in mice (Fig. 3e and f).

### 3.3. 4-PSQ attenuated changes in corticosterone levels caused by Aβ-induction

**Figure 4.**
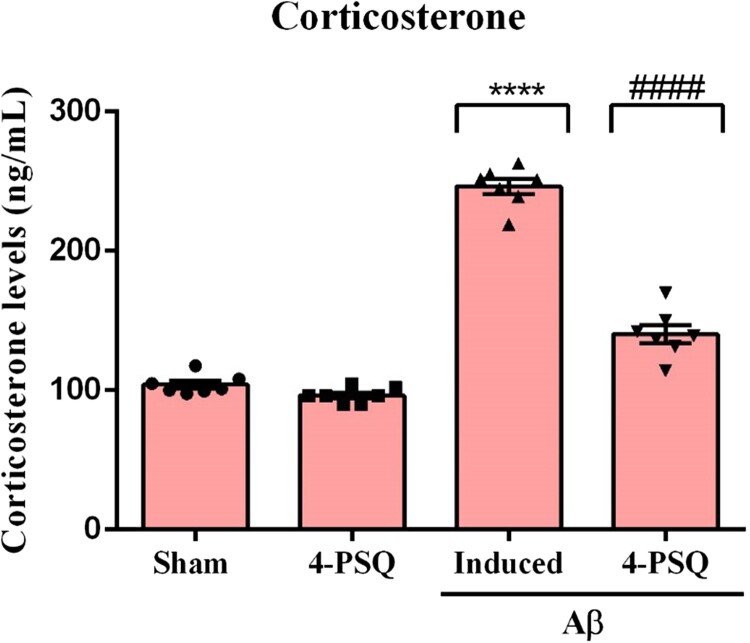
Effect of 7-chloro-4- (phenylselanyl) quinoline (4-PSQ) on corticosterone plasma levels in mice submitted to induction with amyloid β-peptide (Aβ). Values are expressed as mean ± standard error of the mean (S.E.M.) (*n* = 7). (****) denotes *p* < 0.0001 when compared to the sham group. (####) denotes *p* < 0.0001 when compared with the Aβ-induced group (One-way ANOVA followed by the Tukey's test).

The one-way ANOVA followed by Tukey's post-hoc test revealed that Aβ increased (around 137%) plasma corticosterone levels of mice, when compared with the Sham group. Treatment with 4-PSQ normalized these levels (Fig. 4). 4-PSQ *per se* did not change the circulating corticosterone levels in mice. (ANOVA: *F*_(3,24)_ = 233.20, *p* < 0.0001).

### 3.4. 4-PSQ reduced oxidative damage in the prefrontal cortices and hippocampus of Aβ-induced mice

**Figure 5.**
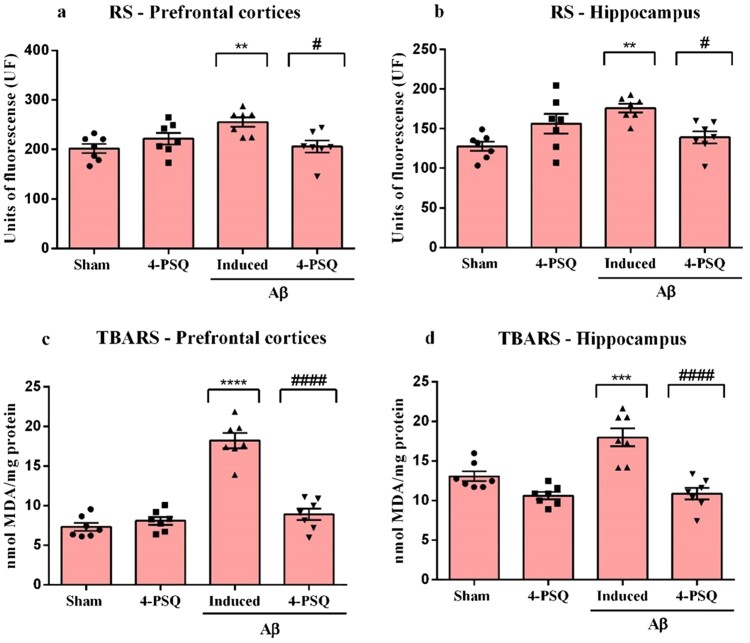
Effect of 7-chloro-4-(phenylselanyl) quinoline (4-PSQ) on markers of oxidative stress in mice submitted to induction with amyloid β-peptide (Aβ). Reactive species (RS) levels in (a) prefrontal cortex and (b) hippocampus; Thiobarbituric acid reactive species (TBARS) levels in (c) prefrontal cortex and (d) hippocampus. Values are expressed as mean ± standard error of the mean (S.E.M.) (*n* = 7). (**) denotes *p* < 0.01, (***) denotes *p* < 0.001 and (****) denotes *p* < 0.0001 when compared to the sham group. (#) denotes *p* < 0.05 and (####) denotes *p* < 0.0001 when compared with the Aβ-induced group (One-way ANOVA followed by the Tukey's test).

Aβ increased a RS level in prefrontal cortices (around 26%) (Fig. 5a) and in hippocampus (around 38%) (Fig. 5b) of mice, when compared with the Sham group. Treatment with 4-PSQ significantly reduced the production of RS caused by Aβ in the cerebral structures. No changes in the RS levels in the prefrontal cortices and hippocampus were observed after *per se* treatment with 4-PSQ. (ANOVA: *F*_(3,24)_ = 5.06, *p* = 0.0074 for the prefrontal cortices and ANOVA: *F*_(3,24)_ = 6.44, *p* = 0.0023 for the hippocampus).

Aβ caused an increase in TBARS levels in prefrontal cortices (around 149%) (Fig. 5c) and in hippocampus (around 37%) (Fig. 5d) of mice, when compared with the Sham group. Treatment with 4-PSQ protected against this increase in cerebral structures of mice. 4-PSQ *per se* did not change the TBARS levels in prefrontal cortices and hippocampus of mice. (ANOVA: *F*_(3,24)_ = 53.82, *p* < 0.0001 for the prefrontal cortices and ANOVA: *F*_(3,24)_ = 18.85, *p* < 0.0001 for the hippocampus).

### 3.5. 4-PSQ modulated the antioxidant enzymes in the prefrontal cortices and hippocampus of Aβ-induced mice

**Figure 6.**
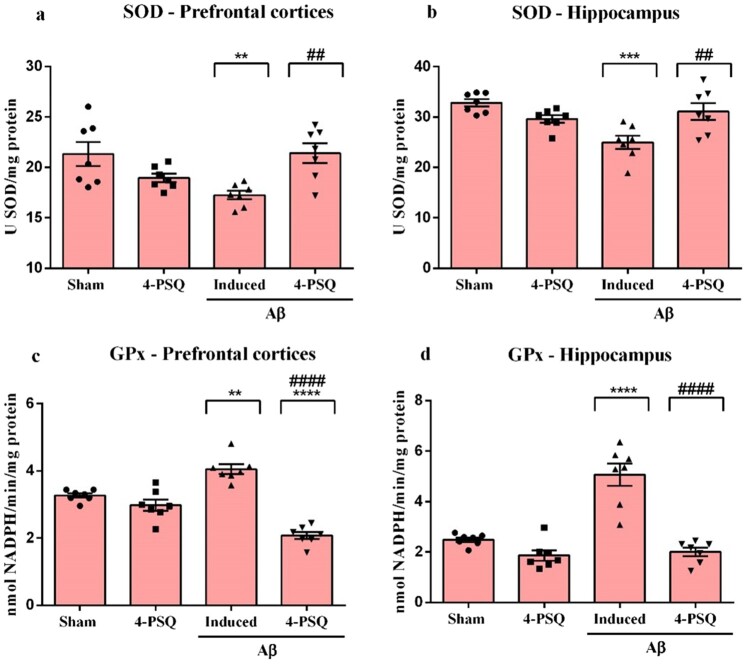
Effect of 7-chloro-4-(phenylselanyl) quinoline (4-PSQ) in the activity of antioxidant enzymes in mice submitted to induction with amyloid β-peptide (Aβ). Superoxide dismutase (SOD) activity in (a) prefrontal cortex and (b) hippocampus; Glutathione peroxidase (GPx) activity in (c) prefrontal cortex and (d) hippocampus. Values are expressed as mean ± standard error of the mean (S.E.M.) (*n* = 7). (**) denotes *p* < 0.01, (***) denotes *p* < 0.001 and (****) denotes *p* < 0.0001 when compared to the sham group. (##) denotes *p* < 0.01 and (####) denotes *p* < 0.0001 when compared with the Aβ-induced group (One-way ANOVA followed by the Tukey's test).

Aβ decreased the SOD activity in prefrontal cortices (around 19%) (Fig. 6a) and in hippocampus (around 24%) (Fig. 6b) of mice, when compared with the Sham group. Treatment with 4-PSQ normalized the SOD activity in the prefrontal cortices and hippocampus of mice. No changes in the SOD activity in the prefrontal cortices and hippocampus were observed after *per se* treatment with 4-PSQ. (ANOVA: *F*_(3,24)_ = 6.003, *p* = 0.0033 for the prefrontal cortices and ANOVA: *F*_(3,24)_ = 8.14, *p* = 0.0007 for the hippocampus).

The one-way ANOVA followed by Tukey's post-hoc test showed that Aβ caused an increase in the GPx activity in prefrontal cortices (around 24%) (Fig. 6c) and in hippocampus (around 104%) (Fig. 6d) of mice, when compared with the Sham group. Treatments with 4-PSQ normalized the activity of this enzyme in prefrontal cortices and hippocampus of the mice. 4-PSQ *per se* did not change the GPx activity in cerebral structures of mice. (ANOVA: *F*_(3,24)_ = 41.18, *p* < 0.0001 for the prefrontal cortices and ANOVA: *F*_(3,24)_ = 33.23, *p* < 0.0001 for the hippocampus).

### 3.6. 4-PSQ reduced levels of TNF-α and IL-6 in the prefrontal cortices and hippocampus of Aβ-induced mice

**Figure 7.**
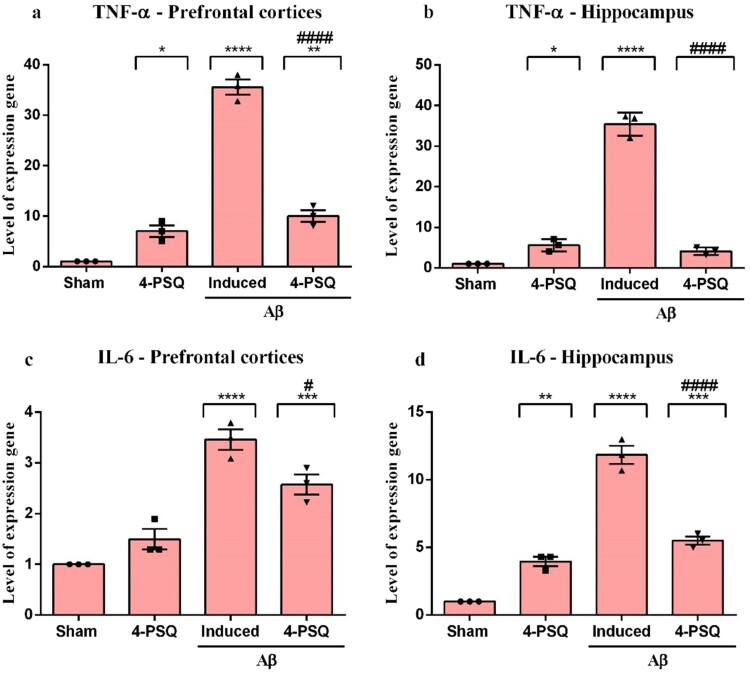
Effect of 7-chloro-4-(phenylselanyl) quinoline (4-PSQ) on the levels of pro-inflammatory cytokines in the brain regions of mice submitted to induction with amyloid β-peptide (Aβ). Tumor necrosis factor alpha (TNF-α) expression in (a) prefrontal cortex and (b) hippocampus; Interleukin-6 (IL-6) expression in (c) prefrontal cortex and (d) hippocampus. Values are expressed as mean ± standard error of the mean (S.E.M.) (*n* = 3). (*) denotes *p* < 0.05, (**) denotes *p* < 0.01, (***) denotes *p* < 0.001 and (****) denotes *p* < 0.0001 when compared to the sham group. (#) denotes *p* < 0.05 and (####) denotes *p* < 0.0001 when compared with the Aβ-induced group (One-way ANOVA followed by the Tukey's test).

Aβ increased the levels of TNF-α and IL-6 in prefrontal cortices (around 340% and 246%, respectively) (Fig. 7a and c) and in hippocampus (around 340% and 385%, respectively) (Fig. 7b and d) of mice, when compared with the Sham group. Treatment with 4-PSQ protected against the increase caused by Aβ induction in mice cerebral structures. 4-PSQ reduced *per se* the levels of inflammatory cytokines in the evaluated structures, except in the prefrontal cortices (in the case of IL-6), when compared with the Sham group. (TNF-α – ANOVA: F_(3,8)_ = 188.00, *p* < 0.0001 for the prefrontal cortices and ANOVA: *F*_(3,8)_ = 270.30, *p* < 0.0001 for the hippocampus); (IL-6 – ANOVA: *F*_(3,8)_ = 40.32, *p* < 0.0001 for the prefrontal cortices and ANOVA: *F*_(3,8)_ = 132.00, *p* < 0.0001 for the hippocampus).

### 3.7. 4-PSQ protected against the increase in GFAP levels Aβ-induced mice

**Figure 8.**
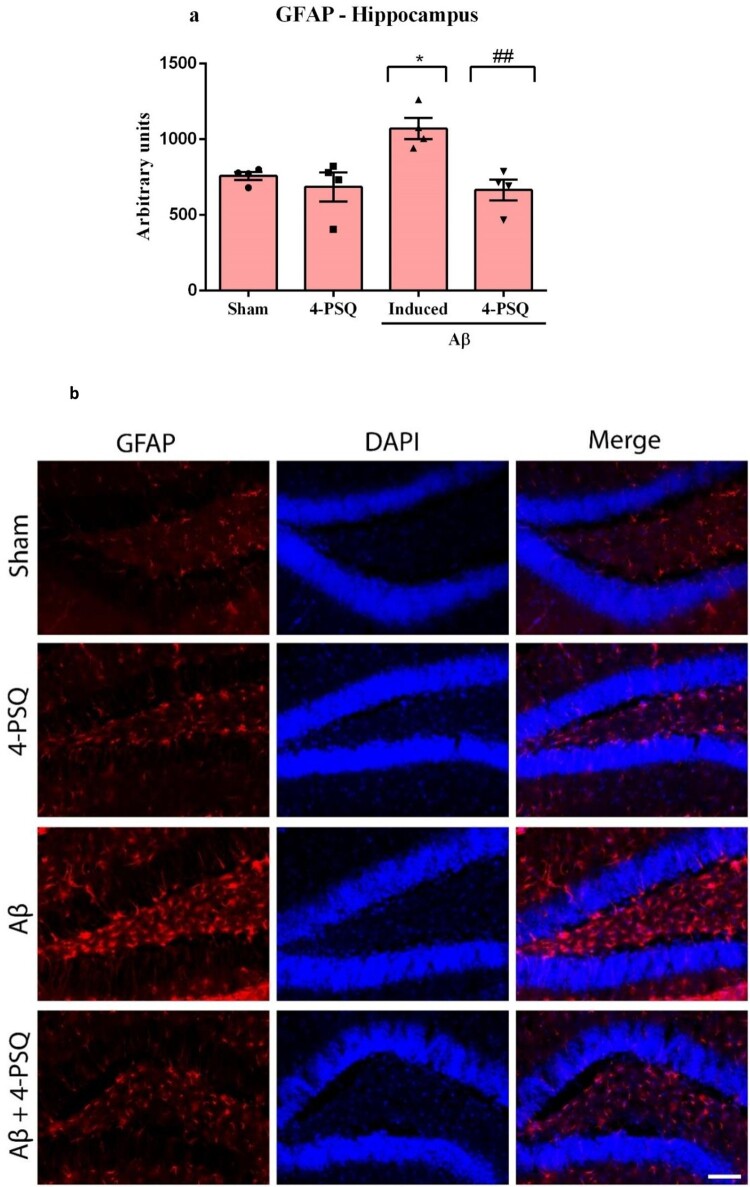
Effect of 7-chloro-4-(phenylselanyl) quinoline (4-PSQ) on amyloid β-peptide (Aβ)-induced alterations in (a) glial fibrillary acidic protein (GFAP) levels in the hippocampus and (b) representative immunofluorescence images. GFAP-positive astrocytes are shown in red (Alexa Fluor 594), and nuclei are counterstained in blue with DAPI. Scale bar: 25 μm. Data are expressed as mean ± standard error of the mean (SEM) (*n* = 4). *p < 0.05 compared with the sham group; ##*p* < 0.01 compared with the Aβ group (one-way ANOVA followed by Tukey’s post hoc test).

The one-way ANOVA followed by Tukey's post-hoc test showed that Aβ caused an increase in the GFAP levels (around 41%) in the hippocampus of mice (Fig. 8a and b), when compared with the Sham group. Treatment with 4-PSQ were effective in protecting against this change. 4-PSQ *per se* did not change the GFAP levels. (ANOVA: *F*_(3,12)_ = 7.32, *p* = 0.0048).

### 3.8. 4-PSQ modulated the AChE activity in the prefrontal cortices and hippocampus of Aβ-induced mice

**Figure 9.**
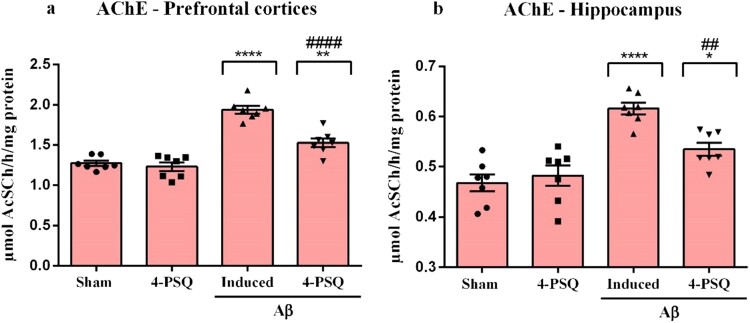
Effects of 7-chloro-4-(phenylselanyl) quinoline (4-PSQ) in the activity of acetylcholinesterase (AChE) in (a) prefrontal cortex and (b) hippocampus in mice submitted to induction with amyloid β-peptide (Aβ). Values are expressed as mean ± standard error of the mean (S.E.M.) (*n* = 7). (*) denotes *p* < 0.05, (**) denotes *p* < 0.01 and (****) denotes *p* < 0.0001 when compared to the sham group. (##) denotes *p* < 0.01 and (####) denotes *p* < 0.0001 when compared with the Aβ-induced group (One-way ANOVA followed by the Tukey's test).

The results showed that Aβ increased the AChE activity in prefrontal cortices (around 52%) (Fig. 9a) and in hippocampus (around 32%) (Fig. 9b) of mice, when compared with the Sham group. This increase was normalized through treatment with 4-PSQ in the cerebral structures of mice. No changes in the AChE activity in the prefrontal cortices and hippocampus were observed after *per se* treatment with 4-PSQ. (ANOVA: *F*_(3,24)_ = 46.82, *p* < 0.0001 for the prefrontal cortices and ANOVA: *F*_(3,24)_ = 18.13, *p* < 0.0001 for the hippocampus).

## 4. Discussion

The high comorbidity between depression and AD, combined with the limited efficacy and adverse effects of current pharmacotherapies, underscores the need for new multi-target agents capable of addressing both cognitive and emotional dysfunction. In this context, the present study demonstrates, for the first time, that 4-PSQ, exerts antidepressant-like and neuroprotective effects in Aβ-induced mice. The behavioral and biochemical data suggest that 4-PSQ acts through modulation of the HPA axis, restoration of redox balance, attenuation of neuroinflammation, and regulation of cholinergic activity.

Consistent with previous studies, Aβ (25–35) administration produced memory impairment and depressive-like behavior in mice [12,30]. 4-PSQ effectively reversed these effects, improving performance in memory and depressive-like behavior tasks, while paroxetine (antidepressant used in clinic) failed to prevent cognitive deficits. The lack of changes in locomotor activity excludes nonspecific psychostimulant or sedative effects. These findings confirm the dual antidepressant-like and pro-cognitive actions of 4-PSQ and highlight its superiority over standard drugs in this comorbidity model.

Hyperactivation of the HPA axis and increased glucocorticoid (GC) release are key contributors to stress-related neuronal dysfunction and are observed in both AD and depression [31]. In the present study, Aβ-induced corticosterone elevation was normalized by 4-PSQ, indicating restoration of HPA axis regulation. Given that chronic GC exposure amplifies oxidative stress and neuroinflammation [32,33], the ability of 4-PSQ to modulate this axis may be a central mechanism in its neuroprotective profile.

Aβ toxicity triggers excessive generation of RS and lipid peroxidation, compromising mitochondrial function, disrupting redox homeostasis, and leading to neuronal death [34,35]. Mitochondrial redox imbalance is an early hallmark of AD, associated with impaired ATP synthesis, calcium dysregulation, and activation of apoptotic pathways [36]. Consistently, Aβ increased RS and TBARS levels in the hippocampus and prefrontal cortex, while 4-PSQ markedly attenuated these effects, confirming its redox-active antioxidant potential. It is believed, the selenium atom in 4-PSQ enables reversible cycling between selenol and selenenic intermediates, scavenging peroxides and maintaining thiol–disulfide balance [10,37]. This catalytic activity mimics that of GPx and thioredoxin reductase, critical for neuronal redox buffering and ferroptosis prevention [9,10]. Thus, 4-PSQ likely acts as a selenoenzyme-mimetic compound reinforcing the antioxidant defense network against Aβ-induced oxidative burden.

Consistent with this mechanism, Aβ decreased SOD and increased GPx activity, an inadequate compensatory response reflecting redox disequilibrium [35]. 4-PSQ normalized both enzymes, re-establishing redox homeostasis and preserving mitochondrial defense. Restoring SOD–GPx balance prevents excessive hydrogen peroxide accumulation and redox amplification, protecting neuronal integrity. These data align with evidence that selenium-based molecules enhance redox resilience by supporting endogenous thiol-dependent antioxidant systems [9,10]. Altogether, these findings suggest that the antidepressant-like and cognitive effects of 4-PSQ derive, at least in part, from its ability to fine-tune redox signaling in brain regions vulnerable to AD pathology.

Neuroinflammation further amplifies Aβ-induced neurotoxicity. Aβ accumulation promotes reactive gliosis, astrocytic hypertrophy, microglial activation, and upregulation of GFAP – accompanied by elevated TNF-α and IL-6 [38,39]. This inflammatory milieu intensifies oxidative stress through redox-sensitive cascades such as NF-κB and Nrf2 dysregulation [40]. In our study, 4-PSQ reduced GFAP expression and TNF-α and IL-6 levels in both regions, demonstrating strong anti-inflammatory action. These effects are consistent with the redox-regulatory capacity of selenium compounds to suppress cytokine release and limit RS-driven glial activation [9,10]. The anti-inflammatory activity of 4-PSQ likely arises from its redox modulation and normalization of HPA axis activity, as both oxidative imbalance and GC excess potentiate neuroimmune signaling [16,17,41]. Together, these findings reinforce the interplay between redox homeostasis and inflammation as a core mechanism of 4-PSQ neuroprotective efficacy.

Dysregulation of cholinergic neurotransmission is a hallmark of AD-related cognitive decline [42]. Excessive AChE activity lowers synaptic acetylcholine and promotes Aβ aggregation, aggravating neuronal dysfunction [43]. Consistent with these mechanisms, Aβ exposure increased AChE activity in the hippocampus and prefrontal cortex, whereas 4-PSQ normalized these alterations. This anticholinesterase effect, in line with previous findings [12,44], may underlie improvements in cognition and mood. Selenium-containing compounds can form redox-active intermediates that influence cholinergic targets and acetylcholine turnover [9], a property likely contributing to 4-PSQ dual therapeutic action.

Taken together, our findings demonstrate that 4-PSQ exerts a multifaceted neuroprotective profile integrating antioxidant, anti-inflammatory, neuroendocrine, and cholinergic modulation. by reducing oxidative stress, suppressing pro-inflammatory cytokines, normalizing GC levels, and restoring cholinergic function, 4-PSQ counteracts the redox and neuroimmune disturbances underlying AD and depression-like comorbidity. Its selenium-centered redox activity appears to coordinate these mechanisms, conferring protection against β-amyloid-induced cognitive and emotional deficits. Altogether, 4-PSQ emerges as a redox-active multitarget compound and promising therapeutic candidate for oxidative stress–related neurodegenerative and neuropsychiatric disorders.

## Supplementary Material

Supplementary_Materials_de_Oliveira_Redox_Letter_clean.docx

## Data Availability

Data supporting the findings of this study are available from the corresponding author upon reasonable request.
