# Supplementary material for: Redox modulation contributes to the antidepressant-like and neuroprotective effects of 7-chloro-4-(phenylselanyl)quinoline in an Alzheimer’s disease model
Source: Redox Rep. 2026 Feb 16;31(1):2626641. doi: 10.1080/13510002.2026.2626641 (PMC12912235; doi:10.1080/13510002.2026.2626641)
Supplement: Supplementary_Materials_de_Oliveira_Redox_Letter_clean.docx [file YRER_A_2626641_SM7008.docx]

**SUPPLEMENTAL DATA**

1. **Supplementary *Materials and methods***
   1. *Animals and ethical approval*

The experiments were conducted using male Swiss mice (25-35g- 60 days old), from a local breeding colony were used. The animals were housed in cages with free access to food and water in a room maintained at 25 ± 1°C with a relative humidity of 50 ± 5%. They were kept on a 12 h light/12 h dark cycle. The experiments were performed according to the guidelines of the Committee on Care and Use of Experimental Animal Resources, Federal University of Pelotas, Brazil (CEEA 1974–2016), following the National Institutes of Health guide for the care and use of laboratory animals (NIH Publications No. 8023, revised 1978). All behavioral tests were conducted during the light period of the light/dark cycle. Every effort was made to minimize the number of animals used and their discomfort.

- 1. *Chemicals*

The 4-PSQ was prepared according to the literature Duarte et al. [1], through nucleophilic substitution between 7-chloroquinoline and phenylselenolate generated in situ. The chemical purity of the compound (99.9%) was determined by gas chromatography - mass spectrometry (GC/MS). Drugs used as a positive control (paroxetine and donepezil) were obtained commercially. 4-PSQ and positive controls was dissolved in canola oil (a non-polar and inert substance) and administered intragastrically (i.g.) at a dose of 1 mg/kg and at a constant volume of 10 mL/kg body weight. The selected dose of 4-PSQ (1 mg/kg, i.g.) was based on previous studies that reported significant effects and confirmed its safety at the same dose, route, and treatment duration [2–4].

Aβ (fragment 25-35) was obtained from Sigma (St. Louis, MO, USA; cat# A4559)). It was dissolved in sterile filtered water and added by incubation at 37°C for 4 days prior to use. All chemicals, reagents, and solvents were obtained from Sigma-Aldrich (St. Louis, MO, USA) at analytical-grade purity. Positive controls (donepezil and paroxetine) and canola oil were sourced from standard commercial suppliers.

- 1. *Experimental protocol*

Mice were randomly divided into 6 experimental groups (7 animals/group). On the first day of the experimental protocol, thirty minutes before initiating induction, the mice belonging to Sham and Aβ-induced groups received the canola oil (10 ml/kg), animals of 4-PSQ and Aβ + 4-PSQ groups received the compound (1 mg/kg, i.g.), and the mice belonging to Aβ + paroxetine and Aβ + donepezil received the paroxetine (1 mg/kg, i.g.) and donepezil (1 mg/kg, i.g.) via gavage, respectively. After treatments, mice belonging to Aβ-induced, Aβ + 4-PSQ, Aβ + paroxetine and Aβ + donepezil groups received Aβ (fragment 25–35) aggregated form (3 nmol/3 μl/per site, by intracerebroventricular (i.c.v.)) [5]. Sham and 4-PSQ groups received saline (3 μl/per site, i.c.v.). The i.c.v. injection of Aβ or vehicle (saline) was administered using a microsyringe with a 28-gauge stainless-steel needle 3.0 mm long (Hamilton) according to a previous report Haley and Mccormick [6]

All animals were anesthetized with isoflurane before i.c.v. injection. Mice were treated with 4-PSQ, paroxetine, donepezil or canola oil every day, until the end of the experimental protocol. On the fifth-day of the experimental protocol, behavioral tests were initiated. On the seventh-day, after the behavioral assessment, the mice were anesthetized (isoflurane inhalation) before blood collection by cardiac puncture [7]. Subsequently, the animals were euthanized to remove brain structures, such as the prefrontal cortices and hippocampus for biochemical analysis.

- 1. *Behavioral tests*

All behavioral tests were scored by a blinded observer, meaning the researcher was unaware of the treatment each mouse received. The same animals underwent the behavioral tests on alternate days according to the experimental protocol.

- - 1. *Open-field test (OFT)*

The OFT evaluated the general locomotor and exploratory behavior of the mice in order to exclude any psychomotor alterations, after administration with Aβ, 4-PSQ, paroxetine or donepezil, on the sixth-day of the experimental protocol [8]. The open-field was made of plywood (30 cm in height x 45 cm in length x 45 cm in width) and divided by masking tape markers into 09 squares (3 rows of 3). Each animal was placed individually at the center of the apparatus and observed for 4 minutes period to record the locomotor (number of segments crossed with the four paws) and exploratory (expressed by the number of time rearing on the hind limbs) activities.

- - 1. *Tail suspension test (TST)*

The TST was conducted as described by Steru et al.[9], being considered a behavioral parameter used to assess the antidepressant-like effect possible of 4-PSQ, paroxetine and donepezil. On the fifth-day of the experimental protocol, the mice were suspended 50 cm above the ground by an adhesive tape placed approximately 1 cm from the tip of the animals tail. Mice were considered immobile only when they hung passively and completely motionless. Immobility time was manually recorded during a 6 minutes period by an experienced observer. In this test, a decrease in the duration of immobility is an indicative of antidepressant-like effect.

- - 1. *Forced swimming test (FST)*

The FST was conducted using the method described by Porsolt et al.[10], on the sixth-day of the experimental protocol. This test is performed to assess the antidepressant-like effect possible of 4-PSQ, paroxetine and donepezil. In this test, mice were individually forced to swim in an open cylindrical container (10 cm in diameter and 25 cm in height), containing 19 cm of water at 25 ± 1°C. The duration of immobility was scored during a 6 minute period by an experienced observer. Each mouse was considered as immobile when floating motionless or making only those movements necessary to keep its head above water. A decrease in the duration of immobility is an indicative of antidepressant-like effect.

- - 1. *Step-down inhibitory avoidance (SDIAT)*

The STDIAT was conducted using the method described by Sakaguchi et al. [11], with modifications of the intensity of electric shock. This test is performed to evaluate non-spatial long-term memory of animals treated with 4-PSQ, paroxetine or donepezil. On the sixth day of experimental protocol, the training session is held, where each mouse was placed on the platform. When it stepped down and placed its four paws on the grid floor, an electric shock (0.5 mA) was delivered for 2 seconds. The test was performed 24 hours after training (seventh-day), where each mouse was placed again on the platform, and the transfer latency time (i.e., time it took to step down from the platform) (seconds) was measured as in the training session, but no electric shock was delivered. The maximum transfer latency time (seconds) was 300 seconds.

- 1. *Biochemical analysis*
     1. *Tissue processing*

On the seventh day of the experimental protocol, mice were anesthetized with isoflurane and blood samples collected from the heart ventricle, using heparin as anticoagulant to obtain plasma. The plasma was obtained by centrifugation (900 × g) for 15 minutes and used to measure the levels of corticosterone. Then, prefrontal cortices and hippocampus were removed and immediately homogenized in cold 50 mM Tris-HCl, pH 7.4 (1/10, w/v). The homogenates were centrifuged at 900 *x*g at 4°C for 10 minutes and supernatant fractions (S1) were used to determine reactive species (RS) levels, thiobarbituric acid reactive species (TBARS) levels and activity of the enzymes superoxide dismutase (SOD) and glutathione peroxidase (GPx). For the determination of the activity of the enzyme acetylcholinesterase (AChE), brain structures of mice were homogenized in 0.25 M sucrose buffer (1/10, w/v) and centrifuged at 900 *x*g at 4°C for 10 minutes.

- - 1. *Plasma corticosterone level*

The changes in the HPA axis were evaluated through the levels of plasma corticosterone, estimated by the fluorescence method previously described by Zenker and Bernstein [12]. Briefly, corticosterone in plasma aliquot was extracted with chloroform. The tubes were shaken for 15 seconds, centrifuged (5 minutes at 900 *x*g), and the aqueous layer was discarded. Then, 0.1 M NaOH was added to tubes and another round of agitation and centrifugation was performed. Lastly, after the addition of the fluorescence reagent (H_2_SO_4_ and 50% ethanol), samples were agitated and centrifuged (5 minutes at 900 *x*g) and incubated at room temperature for 2 hours. After that, the fluorescence intensity emission was recorded at 540 nm (with 257 nm excitation) and corticosterone levels were expressed ng corticosterone/mL plasma.

- - 1. *Oxidative parameters*

Samples of prefrontal cortices and hippocampus were collected to determine RS, and TBARS levels. These measurements were performed to evaluate the effect of 4-PSQ on the modulation of cerebral oxidative stress. RS levels were used as a marker of oxidative damage. The levels of RS formed in the brain structures were determined by spectrofluorimetric using the dichloro-dihydro-fluorescein diacetate (DCHF-DA) reagent [13]. In order to do it, DCHF-DA (1 mM) was incubated together with the S1 and Tris-HCl buffer (10 mM, pH 7.4). The oxidation of DCHF to fluorescent dichlorofluorescein (DCF) was measured for the intracellular RS detection. The fluorescence intensity was measured with emission at 520 nm and excitation at 488 nm in spectrofluorometer (Shimadzu RF-5301 PC fluorometer) and the results are expressed in units of fluorescence (UF).

TBARS content was used as a marker of lipid peroxidation. TBARS levels were determined as described by Ohkawa et al. [14]. An aliquot of S1 was added to the reaction mixture containing: thiobarbituric acid (0.8 %, w/v), sodium dodecyl sulfate (8.1 %, w/v), and acetic acid (pH 3.4) and incubated at 95°C for 2 hours. The absorbance was measured at 532 nm in a spectrophotometer (Shimadzu RF-5301 PC). Results were reported as nmol malondialdehyde (MDA)/mg protein.

- - 1. *Antioxidant enzymes activity*

Antioxidant enzymes have a high capacity to neutralize the formation of RS, being considered the primary cellular defense system [15], so this study evaluated these neurochemical targets. SOD activity was assayed spectrophotometrically as described by Misra and Fridovich [16]. This method is based on the capacity of SOD to inhibit the autoxidation of epinephrine. Briefly, S1 was diluted 1:10 (v/v) to determine SOD activity. S1 aliquot was added to a 0.05 M Na2CO3 buffer, and the enzymatic reaction was started by adding the epinephrine. The color reaction was measured at 480 nm (Shimadzu RF-5301 PC spectrophotometer). One unit of enzyme was defined as the amount of enzyme required to inhibit the rate of epinephrine autoxidation by 50% at 37°C. Results were expressed as units U SOD/mg protein.

GPx activity was evaluated spectrophotometrically (Shimadzu RF-5301 PC) using the method described by Wendel [17], which involves monitoring the reduction of hydrogen peroxide (H_2_O_2_) in the presence of S1 at 340 nm. S1 was added in a system composed by reduced glutathione (GSH)/ nicotinamide adenine dinucleotide phosphate (NADPH)/GR (reduced glutathione), and the enzymatic reaction was initiated by the addition of H_2_O_2_. In this assay, the enzyme activity is indirectly measured by NADPH decay. H_2_O_2_ is reduced and generates oxidized glutathione (GSSG) from GSH. GSSG is regenerated back to GSH by the GR present in the analysis medium at the expense of NADPH. Enzymatic activity was expressed as nmol NADPH/min/mg protein.

- - 1. *RNA extraction and expression of tumor necrosis factor alpha (TNF-α) and interleukin-6 (IL-6) by real‑time PCR*

The total RNA of hippocampus and prefrontal cortex tissue was isolated immediately after the completion of treatment using TRIZOL® (Invitrogen, Carlsbad, CA, USA), according to the manufacturer’s instructions. Total RNA was treated with RNase-free DNase (Invitrogen, Carlsbad, CA, USA), and RNA concentration was determined using a NanoDrop Lite spectrophotometer (Thermo Scientific, Wilmington, DE, USA). Complementary DNA (cDNA) was synthesized from 0.5 μg of total RNA using Oligo(dT) primers (Thermo Scientific), according to the manufacturer’s instructions.

Real-time PCR was performed using SYBR® Green PCR Master Mix with ROX (Invitrogen, Carlsbad, CA, USA). The primers used were synthesized by Invitrogen (São Paulo, Brazil) and are listed in Table 1. PCR reactions were run in a 7500 Real-Time PCR System (Applied Biosystems, Foster City, CA, USA) under the following cycling conditions: 50°C for 15 min, 95°C for 5 min, followed by 40 cycles of 95°C for 15 s and 60°C for 30 s. A melting curve analysis was performed to confirm amplification specificity. All reactions were performed in duplicate, including no-template controls.

Relative gene expression was calculated using the 2⁻ΔΔCt method, with GAPDH as the endogenous control gene. Results were expressed as relative mRNA levels, as by Giongo et al. [18].

**Table 1.** Primer sequences used.

| **Gene** | **Sequence 5’-3’** |
| --- | --- |
| Glyceraldehyde-3-phosphate dehydrogenase (GAPDH) | F: GGGTGAGGCCGGTGCTGAG  R: TGGGGGTAGGAACACGGAAGG |
| Tumor necrosis factor alpha (TNF-α) | F: TCTTCTCATTCCTGCTTGTGGC  R: CACTTGGTGGTTTGCTACGAC |
| Interleukin (IL)-6 | F: CCAGAAACCGCTATGAAG  R: CACCAGCATCAGTCCCAAGA |

- - 1. *Immunofluorescence assay*

Perfused and fixed brains were sectioned on a microtome (Leica, CM3050S), in the coronal plane, sequentially from the beginning of the hippocampus (14 µm) and collected in 6-well culture plates with 1x PBS buffer. Sections were incubated for free-floating for 2 hours in blocking buffer PBS containing 0.1% (v/v) Triton X-100 (PBS-Tx) and 10% (v/v) normal donkey serum at room temperature. After the blocking step, for the immunofluorescence reaction, the sections were incubated overnight at 4°C with the following primary antibodies: anti- glial fibrillary acidic protein (GFAP) (1:400; Sigma). Subsequently, they were washed 3 times for 10 minutes with 1x PBS buffer and incubated for 2 hours at room temperature with the appropriate secondary antibodies to the primary, including: Alexa fluor (1:1000). After incubation the slices were washed 3 times for 10 minutes with 1x PBS buffer and after were incubated with 5 μg/ml of DAPI (Invitrogen/1:1000) for 5 minutes. The slices were transferred to laminas and analyzed using a Nikon Ti2 fluorescence microscope (Nikon, Tokyo, Japan) which has an image capture system. Quantitative analysis of marked cells was made using the Image J software and results expressed as arbitrary units.

- - 1. *AChE activity*

The AChE activity was measured by a modified method of Ellman et al. [19], using acetylthiocholine (AcSCh) as substrate. The method is based on the formation of the yellow anion, 5,50-dithio-bis-acid-nitrobenzoic, measured by absorbance at 412 nm during. Results are expressed as µmol/AcSCh/h/mg protein.

- - 1. *Protein determination*

The protein concentration was measured by the method of Bradford [20], using bovine serum albumin as the standard.

- 1. *Statistical analysis*

Data normality was assessed using the D’Agostino–Pearson test. All datasets showed normal distribution; therefore, parametric comparisons among groups were performed using one-way analysis of variance (ANOVA), followed by Tukey’s post hoc test. Results are presented as mean ± SEM, and statistical significance was set at p < 0.05. Analyses were conducted using GraphPad Prism (GraphPad Software, San Diego, CA, USA).

1. **Reagents, Antibodies, Dyes, and Kits Used in the Study**

**Table 2.** Supplementary Table – Reagents, Antibodies, Dyes, and Kits Used in the Study

| Item | Manufacturer | Catalog Number |
| --- | --- | --- |
| Aβ (25–35) peptide | Sigma-Aldrich | A4559 |
| DCFH-DA (oxidative stress assay) | Sigma-Aldrich | D6883 |
| Thiobarbituric acid (TBA) | Sigma-Aldrich | T5500 |
| Sodium dodecyl sulfate (SDS) | Sigma-Aldrich | L3771 |
| Acetic acid (glacial) | Sigma-Aldrich | A6283 |
| Epinephrine (for SOD assay) | Sigma-Aldrich | E4642 |
| Hydrogen peroxide (H₂O₂) | Sigma-Aldrich | H1009 |
| Reduced glutathione (GSH) | Sigma-Aldrich | G4251 |
| NADPH | Sigma-Aldrich | N7505 |
| TRIzol reagent | Invitrogen (Carlsbad, CA, USA) | 15596026 |
| RNase-free DNase | Invitrogen | 79254 |
| SYBR Green One-Step qRT-PCR Kit with ROX | Invitrogen | 11746100 |
| Oligo(dT) primer | Thermo Scientific | 18418012 |
| Anti-GFAP primary antibody | Sigma-Aldrich | G3893 |
| Alexa Fluor–conjugated secondary antibody | Invitrogen | A-21202 |
| DAPI (nuclear dye) | Invitrogen | D1306 |
| Acetylthiocholine iodide (for AChE assay) | Sigma-Aldrich | A5751 |
| DTNB (Ellman’s reagent) | Sigma-Aldrich | D8130 |
| Isoflurane | Cristália (Brazil) | Without catalog |
| Paroxetine | Obtained commercially | Without catalog |
| Donepezil | Obtained commercially | Without catalog |
| Canola oil | Obtained commercially | Without catalog |

1. **Supplementary References**

[1] Duarte, L. F. B.; Barbosa, E. S.; Oliveira, R. L.; Pinz, M. P.; Godoi, B.; Schumacher, R. F.; Luchese, C.; Wilhelm, E. A.; Alves, D. A Simple Method for the Synthesis of 4-Arylselanyl-7-Chloroquinolines Used as in Vitro Acetylcholinesterase Inhibitors and in Vivo Memory Improvement. *Tetrahedron Lett*, 2017, *58* (33), 3319–3322. https://doi.org/10.1016/j.tetlet.2017.07.039.

[2] Pinz, M.; Reis, A. S.; Duarte, V.; Da Rocha, M. J.; Goldani, B. S.; Alves, D.; Savegnago, L.; Luchese, C.; Wilhelm, E. A. 4-Phenylselenyl-7-Chloroquinoline, a New Quinoline Derivative Containing Selenium, Has Potential Antinociceptive and Anti-Inflammatory Actions. *Eur J Pharmacol*, 2016, *780*, 122–128. https://doi.org/10.1016/j.ejphar.2016.03.039.

[3] Pinz, M. P.; Reis, A. S.; Vogt, A. G.; Krüger, R.; Alves, D.; Jesse, C. R.; Roman, S. S.; Soares, M. P.; Wilhelm, E. A.; Luchese, C. Current Advances of Pharmacological Properties of 7-Chloro-4-(Phenylselanyl) Quinoline: Prevention of Cognitive Deficit and Anxiety in Alzheimer’s Disease Model. *Biomedicine and Pharmacotherapy*, 2018, *105* (June), 1006–1014. https://doi.org/10.1016/j.biopha.2018.06.049.

[4] Paltian, J. J.; dos Reis, A. S.; de Oliveira, R. L.; da Fonseca, C. A. R.; Domingues, W. B.; Dellagostin, E. N.; Campos, V. F.; Kruger, R.; Alves, D.; Luchese, C.; et al. The Anxiolytic Effect of a Promising Quinoline Containing Selenium with the Contribution of the Serotonergic and GABAergic Pathways: Modulation of Parameters Associated with Anxiety in Mice. *Behavioural Brain Research*, 2020, *393*, 112797. https://doi.org/10.1016/j.bbr.2020.112797.

[5] Ianiski, F. R.; Alves, C. B.; Souza, A. C. G.; Pinton, S.; Roman, S. S.; Rhoden, C. R. B.; Alves, M. P.; Luchese, C. Protective Effect of Meloxicam-Loaded Nanocapsules against Amyloid-β Peptide-Induced Damage in Mice. *Behavioural Brain Research*, 2012, *230* (1), 100–107. https://doi.org/10.1016/j.bbr.2012.01.055.

[6] HALEY, T. J.; McCORMICK, W. G. PHARMACOLOGICAL EFFECTS PRODUCED BY INTRACEREBRAL INJECTION OF DRUGS IN THE CONSCIOUS MOUSE. *Br J Pharmacol Chemother*, 1957, *12* (1), 12–15. https://doi.org/10.1111/j.1476-5381.1957.tb01354.x.

[7] Parasuraman, S.; Raveendran, R.; Kesavan, R. Blood Sample Collection in Small Laboratory Animals. *J Pharmacol Pharmacother*, 2010, *1* (2), 87–93. https://doi.org/10.4103/0976-500X.72350.

[8] Walsh, R. N.; Cummins, R. A. The Open-Field Test: A Critical Review. *Psychol Bull*, 1976, *83* (3), 482–504. https://doi.org/10.1037/0033-2909.83.3.482.

[9] Steru, L.; Chermat, R.; Thierry, B.; Simon, P. The Tail Suspension Test: A New Method for Screening Antidepressants in Mice. *Psychopharmacology (Berl)*, 1985, *85* (3), 367–370. https://doi.org/10.1007/BF00428203.

[10] Porsolt, R. D.; Le Pichon, M.; Jalfre, M. Depression: A New Animal Model Sensitive to Antidepressant Treatments. *Nature*, 1977, *266* (5604), 730—732. https://doi.org/10.1038/266730a0.

[11] Sakaguchi, M.; Koseki, M.; Wakamatsu, M.; Matsumura, E. Effects of Systemic Administration of β-Casomorphin-5 on Learning and Memory in Mice. *Eur J Pharmacol*, 2006, *530* (1–2), 81–87. https://doi.org/10.1016/j.ejphar.2005.11.014.

[12] Zenker, N.; Bernstein, D. E. The Estimation of Small Amounts of Corticosterone in Rat Plasma. *J Biol Chem*, 1958, *231* (2), 695–701. https://doi.org/10.1016/s0021-9258(18)70434-1.

[13] Loetchutinat, C.; Kothan, S.; Dechsupa, S.; Meesungnoen, J.; Jay-Gerin, J. P.; Mankhetkorn, S. Spectrofluorometric Determination of Intracellular Levels of Reactive Oxygen Species in Drug-Sensitive and Drug-Resistant Cancer Cells Using the 2′,7′-Dichlorofluorescein Diacetate Assay. *Radiation Physics and Chemistry*, 2005, *72* (2–3), 323–331. https://doi.org/10.1016/j.radphyschem.2004.06.011.

[14] Ohkawa, H.; Ohishi, N.; Yagi, K. Assay for Lipid Peroxides in Animal Tissues by Thiobarbituric Acid Reaction. *Anal Biochem*, 1979, *95* (2), 351–358. https://doi.org/10.1016/0003-2697(79)90738-3.

[15] Muller, F. L.; Song, W.; Liu, Y.; Chaudhuri, A.; Pieke-Dahl, S.; Strong, R.; Huang, T. T.; Epstein, C. J.; Roberts, L. J.; Csete, M.; et al. Absence of CuZn Superoxide Dismutase Leads to Elevated Oxidative Stress and Acceleration of Age-Dependent Skeletal Muscle Atrophy. *Free Radic Biol Med*, 2006, *40* (11), 1993–2004. https://doi.org/10.1016/j.freeradbiomed.2006.01.036.

[16] Misra, H. P.; Fridovich, I. The Role of Superoxide Anion in the Autoxidation of Epinephrine and a Simple Assay for Superoxide Dismutase. *J Biol Chem*, 1972, *247* (10), 3170—3175.

[17] Wendel, A. Glutathione Peroxidase. *Methods Enzymol*, 1981, *77* (C), 325–333. https://doi.org/10.1016/S0076-6879(81)77046-0.

[18] Giongo, J. L.; de Almeida Vaucher, R.; Sagrillo, M. R.; Vianna Santos, R. C.; Duarte, M. M. M. F.; Rech, V. C.; Soares Lopes, L. Q.; Beatriz da Cruz, I.; Tatsch, E.; Moresco, R. N.; et al. Anti-Inflammatory Effect of Geranium Nanoemulsion Macrophages Induced with Soluble Protein of Candida Albicans. *Microb Pathog*, 2017, *110*, 694–702. https://doi.org/10.1016/j.micpath.2017.01.056.

[19] Ellman, G. L. Tissue Sulfhydryl Groups. *Arch Biochem Biophys*, 1959, *82* (1), 70–77. https://doi.org/10.1016/0003-9861(59)90090-6.

[20] Bradford, M. M. A Rapid and Sensitive Method for the Quantitation of Microgram Quantities of Protein Utilizing the Principle of Protein-Dye Binding. *Anal Biochem*, 1976, *72* (1–2), 248–254. https://doi.org/10.1016/0003-2697(76)90527-3.
